# Plasma Lactate Response to a 12‐Min Walk Test Is a Poor Diagnostic Biomarker for Mitochondrial Myopathy

**DOI:** 10.1002/jmd2.70112

**Published:** 2026-08-27

**Authors:** Christine Lando, Tahmina Khawajazada, Jesper Helbo Storgaard, Nanna Scharff Poulsen, John Vissing, Nicoline Løkken

**Affiliations:** ^1^ Copenhagen Neuromuscular Center, Department of Neurology Copenhagen University Hospital, Rigshospitalet Copenhagen Denmark; ^2^ Department of Clinical Medicine University of Copenhagen Copenhagen Denmark

**Keywords:** 12‐min walk test, 6‐min walk test, diagnostic screening test, mitochondrial myopathy, plasma lactate, relative workload

## Abstract

Mitochondrial myopathy (MM) represents a group with a broad phenotypic spectrum which complicates the diagnostic process. This study investigated whether plasma lactate changes following a 12‐min walk test (12MWT) and 20‐min recovery could be a diagnostic screening tool for MM. Thirty patients with MM and 19 healthy controls (HC) participated. Total walking distance and heart rate during the test were measured, along with plasma lactate at rest, after exercise, and during recovery. For the latter, we calculated the area under the curve (AUC). The results were compared between groups. MM patients walked shorter (1082 m [994–1143] m vs. 1349 m [1149–1435], *p* = 0.0005) and had higher resting lactate (1.7 ± 1.2 mmol/L vs. 0.9 ± 0.3 mmol/L, *p* = 0.003). No significant difference in plasma lactate was observed after exercise. Plasma lactate AUC during recovery was similar between groups. Thus, the 12MWT is not a reliable screening tool for MM as changes in plasma lactate during exercise and recovery did not differentiate MM from HC.

## Key Points

- The 12‐min walk test is not a reliable diagnostic screening test for mitochondrial myopathy.
- Plasma lactate responses to a 12‐min walk test did not reliably distinguish patients with mitochondrial myopathy from healthy controls.
- Resting lactate was higher in patients with mitochondrial myopathy compared to healthy controls.
- The 12‐min walk test may serve as a functional outcome measure, but further studies are needed to establish its validity and reliability.

**Abbreviations**
12MWT: 12‐min walk test
6MWT: 6‐min walk test
AUC: area under the curve
HC: healthy controls
MM: mitochondrial myopathy
mtDNA: mitochondrial DNA

## 1 Introduction

In primary mitochondrial disease, genetic defects in mitochondrial or nuclear DNA lead to mitochondrial dysfunction. These conditions can present with complex symptoms, predominantly impacting high‐energy‐demanding organs, such as the central nervous system, cardiac muscle tissue, and skeletal muscles. It is termed mitochondrial myopathy (MM) when symptoms primarily involve the skeletal muscles. MM constitutes a diverse group with early muscle fatigue, general weakness, and exercise intolerance as the most common manifestations. Diagnosis of individuals with MM is often delayed due to the broad phenotypic spectrum and the dependence on invasive, complicated, and expensive diagnostic tests, resulting in many individuals remaining undiagnosed for prolonged periods [1]. The current diagnostics involve genetic testing using next‐generation sequencing (NGS) and muscle biopsy evaluation [2, 3]. Although NGS is sensitive, it is not always readily accessible, and patients often present with nonspecific symptoms or variants. Clinically feasible functional assessments therefore remain valuable for prioritizing patients for molecular testing and for physiologically validating variants of uncertain significance (VUS). These challenges call for simpler, less invasive, yet sensitive and specific screening tests for MM. In particular, exercise‐based screening approaches have been pursued. Several protocols have been suggested for single‐extremity exercise, like the handgrip test. These studies assess the oxygen extraction by the working muscle or plasma lactate production in MM patients. The deoxygenation protocols show varying results with a range of 21%–92% for sensitivity and 91%–100% for specificity [4, 5, 6], and the plasma lactate measurements have been deemed not diagnostically useful both in 3‐ and 6‐min handgrip tests [5, 6, 7]. Different protocols for measuring plasma lactate and deoxygenation during bicycle ergometry have also been studied. These studies use either a constant relative workload [8, 9, 10], a constant absolute workload [10, 11, 12, 13], or an incremental protocol until exhaustion [8, 14, 15]. The studies' results show diagnostic sensitivity ranging from 27% to 88% and specificity ranging from 84% to 100%. However, the common tendency in these cycling studies is a high specificity but a low sensitivity [8, 9, 10, 11, 12, 13]. Certain studies favor lactate stress testing for its higher sensitivity rather than resting lactate determination. This is because MM patients may exhibit normal resting lactate levels [13, 15] and because their lactate recovery can be prolonged compared to healthy controls (HC) [8].

Mitochondrial function is compromised to a similar degree in both MM patients and HC at maximal exertion, rendering a lactate stress test during peak exercise ineffective for diagnostics. Therefore, the most common aerobic activity in daily life, that is, walking, could be considered an alternative to bicycle ergometry testing, and it has yet to be investigated in MM patients. The 6‐min walk test (6MWT) is a simple test that can be conducted almost anywhere, and it is a well‐described tool for assessing patients with neuromuscular diseases. A 6MWT might not be suitable for MM patients, as they may not reach the lactate threshold within this time frame. Consequently, we aimed to examine whether plasma lactate responses following a 12‐min walk test (12MWT) and a 20‐min recovery period could serve as a potential diagnostic screening test for MM versus HC. Additionally, we aimed to compare these responses to those from a 6MWT and assess the effect of fasting on lactate levels.

## 2 Methods

### 2.1 Participants

We included 30 patients with MM and 19 HC who were similar to the MM patient group in regards to age and sex. Inclusion criteria were age between 18 and 75 years and genetically verified MM if not HC. Exclusion criteria were the presence of confounding diseases and the inability to complete a walk test. The HC were recruited via the online platform *forsøgsperson.dk*. Ethical authorization was obtained from the Committee on Health Research Ethics of the Capital Region of Denmark (approval no. H‐17032512). Preceding inclusion, the trial was registered on ClinicalTrials.gov (NCT03513835). All participants were sufficiently informed of the potential study risks and benefits and gave written consent before participating.

### 2.2 Experimental Procedure

**FIGURE 1:**
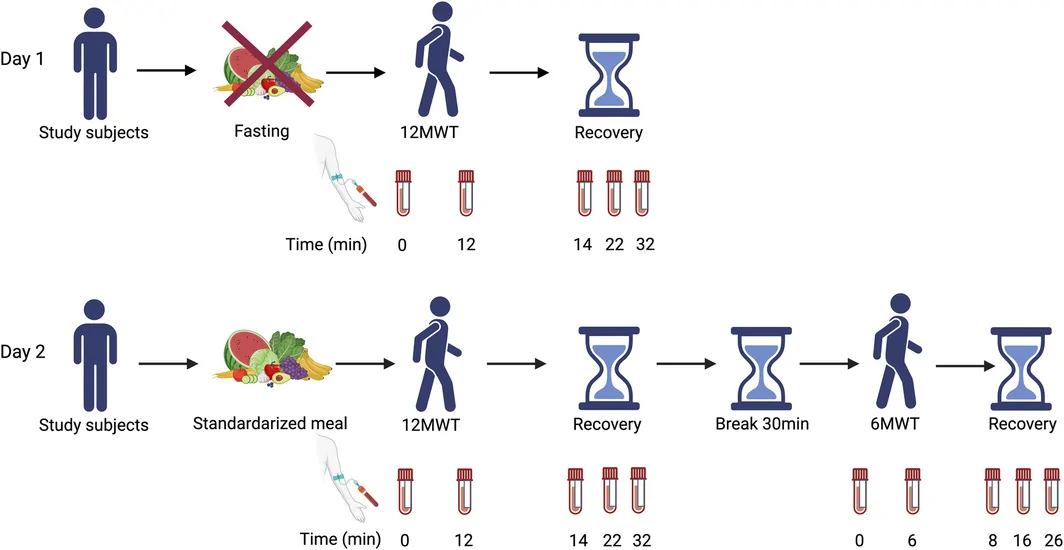
Experimental setup. Schematic representation of the dietary and exercise intervention including timing of blood sample collection (indicated by tubes) on the test days. 12MWT, 12‐min walk test; 6MWT, 6‐min walk test; min, minutes.

All participants performed a 12MWT on two consecutive days, see Figure 1. Subjects were instructed to refrain from alcohol and caffeine consumption for 12 h and exercise for 24 h prior to participation. Participants met fasting. A peripheral venous catheter was placed into the cubital vein of one arm. For the 12MWT, participants were instructed to walk at their maximum pace for 12 min between two markers positioned 30 m apart. Instructions through the test were standardized. Venous blood samples were drawn before the test (T:0 = rest), at the end of the test (T:12 = end), and during the recovery phase at 2, 10, and 20 min posttest (T:14, 22, and 32). Distance was recorded as the number of completed laps during the test and converted to meters. The partial lap at the end of the test was measured using a measuring tape and added to the total walking distance. Pulse was continuously monitored using a pulse belt and watch, with readings recorded every minute. The average heart rate was then calculated from these minute‐by‐minute readings. On the second day of testing, participants repeated the same 12MWT protocol with equivalent blood samples, but this time with a standardized meal given in advance. The aim was to assess whether nutritional status influenced plasma lactate responses during submaximal exercise, as postprandial metabolic conditions may affect lactate production. After completing the walk test and the 20‐min recovery period, subjects rested for an additional 30 min before starting a 6MWT. During the 6MWT, the pulse was monitored, and distance metrics were noted identically to the 12MWT. Blood samples were again drawn before the 6MWT (T:0 = rest), at the end of the test (T:6 = end), and during the recovery phase at 2, 10, and 20 min.

### 2.3 Blood Sampling and Analysis

Heparinized venous blood samples were collected and promptly analyzed for plasma lactate, partial pressure of oxygen (PaO_2_) and carbon dioxide (PaCO_2_), and plasma glucose levels using an ABL90 FLEX blood gas analyzer (Radiometer, Denmark).

### 2.4 Statistical Analysis

We used Rx64 v. 4.3.0 and RStudio software v. 2023.09.0. for statistical tests. Normality and variance were assessed. Continuous variables are presented as mean ± SD or median (IQR), as appropriate. Non‐normal distributed data were analyzed using the non‐parametric Wilcoxon rank‐sum test. Binomial data were compared with the Fisher test. Lactate measures were analyzed using a linear mixed‐effects model with participant‐specific random intercepts to account for within‐subject correlation. Fixed effects included group (MM vs. HC), time, and the group × time interaction. Because lactate concentrations were positively skewed, analyses were performed on log‐transformed lactate values.

A linear regression analysis was conducted to assess differences between groups when including HR as a covariate. To compare the 12MWT lactate recovery between groups, we calculated the lactate area under the curve (AUC) from end of exercise (T:12) to end of recovery (T:32) and compared it with a two‐sample *t*‐test. Significant differences between groups were calculated both with and without outliers and it is noted if it changed the outcome. Statistical significance was defined as a two‐sided *p* < 0.05.

## 3 Results

### 3.1 Participants

Of the 30 MM subjects that completed the first test day, five did not complete the second test day (patients #2, #5, #16, #29, and #30). All 19 HC completed both test days. Patient characteristics are shown in Table 1. Patients (#18 and #19) and (#25, #26, and #27) were siblings, and patients (#29 and #30) were mother and daughter. Two patients had diabetes mellitus, but they were effectively managing it with insulin, and one patient was on anti‐epileptic medication. Among the patients, 26 experienced exercise intolerance and 24 muscle fatigue. We observed no difference in age, BMI, or sex between groups (Table 2).

**TABLE 1:** Patient characteristics and results from the 12MWT performed on test day 1.

No.	Sex/Age	BMI	Phenotype	Genotype	Distance (m)	Avr. HR (bpm)	Muscle mutation load
1	F/64	19.8	CPEO	m.12113_14422del2310bp	495.5	94	50%
2	M/40	23.5	CPEO	m.6391_13186del679bp	1106.9	74	15%–20%
3	F/70	24.0	CPEO	m.9110_14605del	721.9	97	26%–42%
4	M/27	19.3	CPEO	mt8937_13030del4094	1360.5	155	63%
5	M/58	25.1	NARP	m.8989G>C	305.6	105	92%
6	F/56	20.9	MERRF	m.8344A>G	1110.0	111	85%
7	F/27	41.0	MERRF	m.8344A>G	1107.6	130	78%
8	M/54	27.6	CPEO	m.7177_13767del	1028.9	106	51%
9	F/53	33.1	POLG	c.2766G>C	1084.0	134	NA
10	M/61	27.0	MERFF	m.8344A>G	665.0	98	63%
11	M/45	29.4	EI	m.5133_5134delAA	990.0	140	90%–92%
12	F/53	18.9	POLG	c.158_166dup and c.1174C>G	894.0	NA	NA
13	F/56	19.3	CPEO/KSS	m.6098_12201del6104bp	1050.0	79	10%
14	F/59	19.1	POLG	c.251 T>I and c.467A>T	832.8	104	NA
15	M/49	21.3	CPEO	m.13585_15596del	1236.4	96	70%–75%
16	M/40	24.4	CPEO	m.9481_13734del	1060.4	95	50%
17	F/52	26.0	CPEO	≈4000 bp mt deletion	1101.5	112	NA
18	F/46	20.1	MELAS	m.3243A>G	1221.1	146	33%
19	F/41	22.6	MELAS	m.3243A>G	1173.8	130	34%
20	M/59	27.8	CPEO	m.561_3895del	1054.0	103	NA
21	M/47	25.2	CPEO	m.4087A>G	1141.5	150	100%
22	F/26	22.7	CPEO	m.9412_14410del4999bp	1004.0	129	NA
23	F/24	19.3	MELAS	m.3243A>G	1256.0	111	NA
24	M/55	27.2	CPEO/KSS	m.5909_13990del8082bp	1117.8	NA	NA
25	M/30	27.3	MELAS	m.3243A>G	1078.0	NA	NA
26	M/27	26.6	MELAS	m.32a3A>G	1143.7	NA	NA
27	M/24	21.2	MELAS	m.3243A>G	1383.8	127	NA
28	M/39	27.7	Leigh syndr	c.626C>T and c.626C>T	740.0	108	NA
29	F/55	23.3	MELAS	m.3243A>G	1208.0	131	33%
30	F/30	22.1	MELAS	m.3243A>G	1080.0	130	NA

*Note:* Age is presented in years, bpm, beats per minute; CPEO, chronic progressive external ophthalmoplegia; EI, exercise intolerance; F, Female; KSS, Kearns‐Sayres syndrome; M, Male; m, meters; MELAS, mitochondrial encephalopathy lactic acidosis and stroke‐like episodes; MERRF, myoclonic epilepsy with ragged‐red fibers; NA, not available; NARP, neurogenic muscle weakness ataxia, and retinitis pigmentosa; No., patient number; POLG, mutations in the PolG‐gene.

**TABLE 2:** Summary statistics.

	MM		HC		HC vs. MM (*p*‐value)
Sex (M/F)	15/15		11/8		*p* = 0.806
Age (years)	48 [32–56]		47 [36–59]		*p* = 0.681
BMI	24 [21–27]		24 [22–27]		*p* = 0.590
12 MWT	**Day 1 (*n* = 30)**	**Day 2 (*n* = 25)**	**Day 1 (*n* = 19)**	**Day 2 (*n* = 19)**	
Distance (m)	1082 [994–1143]	1110 [1020–1200]	1349 [1149–1435]	1344 [1221–1445]	**Day 1 *p* < 0.0005**
					**Day 2 *p* < 0.0001**
Mean HR (bpm)	111 [99–130]	123 [109–137]	117 [108–137]	131 [120–147]	Day 1 *p* = 0.297
					Day 2 *p* = 0.215
p‐Lac T:0	1.7 ± 1.2	2.5 ± 1.5	0.9 ± 0.3	1.4 ± 0.3	**Day 1 *p* = 0.003**
					**Day 2 *p* = 0.006**
p‐Lac T:12	3.1 ± 2.8	4.2 ± 3.7	2.3 ± 1.3	3.2 ± 2.3	Day 1 *p* = 0.190
					Day 2 *p* = 0.069
p‐Lac T:14	3.4 ± 3.7	4.6 ± 4.0	2.3 ± 1.4	2.9 ± 1.8	
p‐Lac T:22	3.0 ± 3.6	4.3 ± 4.4	1.8 ± 1.1	2.5 ± 1.7	
p‐Lac T:32	2.6 ± 3.3	3.7 ± 3.9	1.3 ± 0.8	2.0 ± 1.2	**Day 1 *p* = 0.010**
					**Day 2 *p* = 0.003**
AUC (T:12–32)	59.7 ± 70.8	84.2 ± 83.3	37.8 ± 22.9	47.5 ± 29.5	Day 1 *p* = 0.132
					Day 2 *p* = 0.052
6 MWT					
Distance (m)		548 [513–620]		690 [630–720]	***p* < 0.0001**
p‐Lac T:0		2.6 ± 2.5		1.2 ± 0.4	***p* = 0.002**
p‐Lac T:6		3.6 ± 3.4		2.5 ± 1.6	*p* = 0.098
p‐Lac T:26		3.2 ± 3.7		1.5 ± 0.8	***p* = 0.002**

*Note:* Data is presented as median [IQR] or mean ± SD. Recovery time points were not analyzed individually because multiple repeated measurements were obtained during recovery. To avoid multiple comparisons, recovery responses were summarized as area under the curve (AUC) and compared. Significant differences between MM and HC are highlighted in bold (*p* < 0.05).

Abbreviations: bpm, beats per minute; F, female; HC, healthy control; HR, heart rate; M, male; m, meter; MM, mitochondrial myopathy; MWT, minute walk test; p‐Lac, plasma lactate concentration (mmol/L); T, time point in minutes from test start.

We observed no difference between groups in plasma glucose levels, PaO_2_, or PaCO_2_; therefore, plasma lactate remained the studied biomarker.

### 3.2 Walking Distance and Heart Rate

**FIGURE 2:**
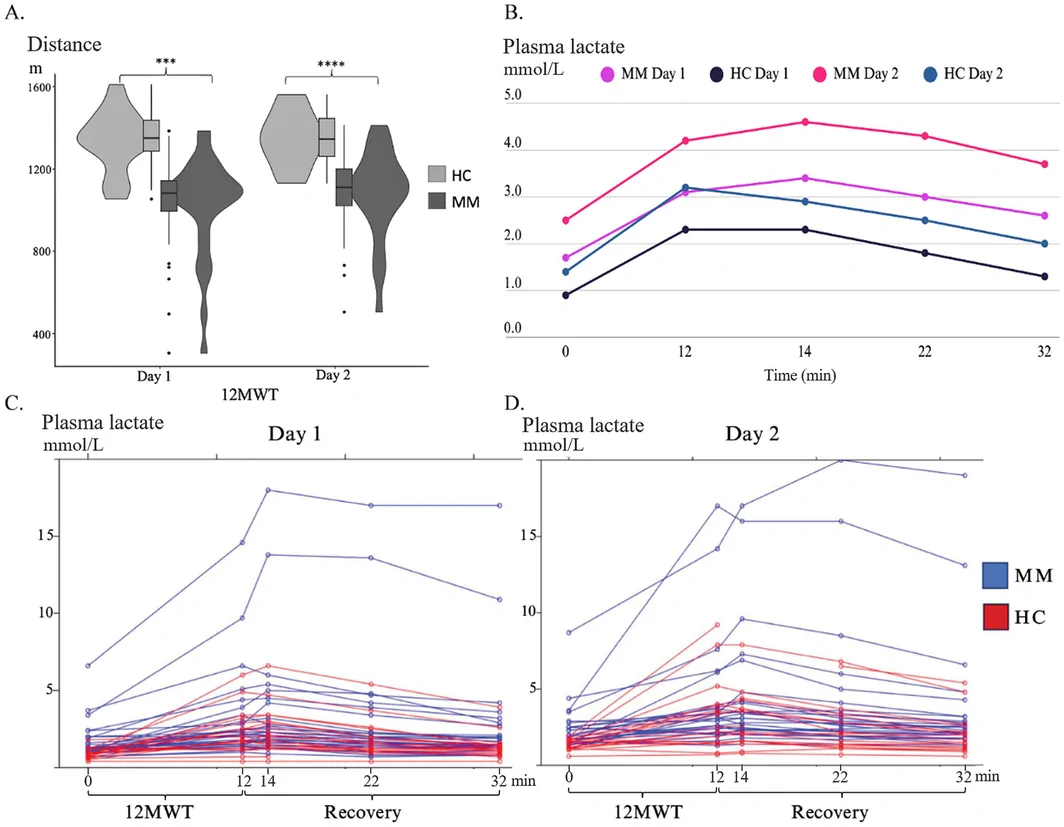
Walking performance and plasma lactate responses during the 12‐min walk test (12MWT). (A) The distance walked on test day 1 and 2 illustrated with violine plots. Plot width reflects data distribution density; boxes indicate the interquartile range, horizontal lines mark the median, and whiskers represent data spread. Outliers are shown as individual points. Statistical significance: ***p < 0.0005, ****p < 0.0001. (B) Mean plasma lactate concentrations and (C, D) individual plasma lactate responses measured at rest (timepoint 0 min), at test completion (timepoint 12 min), and during recovery (12–32 min) in patients with mitochondrial myopathy (MM) and healthy controls (HC), shown for test day 1 and test day 2.

The MM group walked significantly shorter than the HC group on both days of the 12MWT and in the 6MWT. The results are presented in Table 2 and for the 12MWT visualized in Figure 2A. No intragroup differences were observed in the 12MWT distance between the two test days in either group. There was no difference in average HR between the groups (Table 2).

### 3.3 Resting Plasma Lactate

Resting plasma lactate levels were significantly higher in the MM group compared to the HC group on both test days (Table 2 and Figure 2B). In both groups, the resting lactate values differed between the 2 days within groups. There were significantly lower lactate levels when fasting (test day 1) compared to the day of the standardized meal (test day 2) (MM = *p* < 0.0001, HC = *p* < 0.0001).

### 3.4 Plasma Lactate During Exercise and Recovery

Participants' individual plasma lactate response to the 12MWT and post exercise recovery on the two test days are shown in Figure 2C,D, and the mean plasma lactate concentrations are shown in Figure 2B and Table 2. We found no difference in lactate levels immediately after exercise in any of the tests between the two groups.

The plasma lactate recovery was evaluated by calculating the AUC (T:12‐T:32), and we found no difference between the groups in the recovery plasma‐lactate AUC (Table 2). Although the Day 2 comparison approached statistical significance (*p* = 0.052), this finding was highly influenced by the two outliers (patient #4 and #11). After exclusion of these two outliers, the *p*‐value increased to 0.152, indicating that the apparent trend was driven primarily by these individual data points and should therefore be interpreted with caution rather than as evidence of a robust group effect. At end‐of‐recovery, lactate levels had almost returned to resting values in most participants, and consequently the between‐group comparison approached the same significant difference observed before the test at rest (Table 2).

Although there was no statistically significant difference in HR between the groups, we introduced HR as a covariate in a linear regression analysis to account for potential differences in exercise intensity. Across both test days, there was a consistent positive association between mean HR and end‐lactate (T:12), indicating that higher HR was associated with higher lactate levels. This association was statistically significant on both days in the primary models and remained robust across different model specifications (test day 1: SE = 0.017, *p* = 0.001; test day 2: SE = 0.022, *p* < 0.001). Supporting this, correlation analyses showed that mean HR was moderately positively correlated with lactate both at test completion (T:12: Spearman's *ρ* = 0.47, *p* = 0.015) and during recovery (T:32: *ρ* = 0.51, *p* = 0.008), suggesting that cardiovascular strain is an important determinant of lactate response. In addition, mean HR was positively correlated with walking distance (*ρ* = 0.47, *p* = 0.017), indicating that participants with higher exercise intensity tended to cover longer distances. Together, these findings support the inclusion of HR as a covariate.

However, no significant differences in lactate levels were observed between MM and HC after adjustment for HR on either day (test day 1: SE = 0.71, *p* = 0.107; test day 2: SE = 0.99, *p* = 0.16). There was no evidence of an interaction between group and heart rate, suggesting that the relationship between heart rate and lactate was similar in both groups.

### 3.5 Comparison Between 12MWT and 6MWT

When comparing lactate accumulation between 12MWT and 6MWT, we found that MM patients exhibited significantly higher lactate levels at end‐of‐test following the 12MWT than they did following the 6MWT (*p* = 0.004). In contrast, there was no statistically significant difference in end‐of‐test lactate levels between a 12MWT and a 6MWT in the HC group (*p* = 0.142). We did not see any substantial decrease in the cadence of the MM patients in the last 6 min of the 12MWT; the walking pace in both the MM and HC groups remained steady throughout the 12MWT.

## 4 Discussion

### 4.1 Main Findings

The study explored the 12‐min submaximal exercise walk test as a potential diagnostic screening tool for MM. We measured walking distance and plasma lactate at rest, at the end of the test and during a 20‐min post‐exercise recovery period. Our results show that a 12MWT is not a reliable diagnostic screening tool for MM. While the patients walked significantly shorter on both days than HC, this alone is not specific to MM. Plasma lactate was not a useful diagnostic biomarker, as neither end‐exercise lactate nor recovery lactate changes differed between patients with MM and HC. In fact, plasma lactate showed substantial overlap between groups, making it impossible to establish a clinically meaningful diagnostic lactate threshold. Consequently, sensitivity and specificity analyses were not meaningful.

### 4.2 Resting versus Exercise Lactate

Resting plasma lactate was significantly higher in patients with MM compared with HC. Some studies recommend lactate stress testing for higher sensitivity than resting lactate determination in MM [8, 13]. Our results, however, show no significant difference in plasma lactate between MM and HC after submaximal exercise or during recovery. In contrast, exercise reduced the difference in plasma lactate between the two groups rather than increasing it.

### 4.3 Exercise Intensity and Relative Effort

We speculated that an extended exercise duration of the more commonly used 6MWT would stress mitochondrial metabolism more, revealing group differences, but this was not the case. While MM patients walked significantly shorter distances in the 6MWT, the 12MWT did not produce a larger between‐group difference in plasma lactate than the 6MWT. Instead, the results suggest a group‐dependent response, with differences in end‐exercise lactate between 12MWT and 6MWT evident in MM patients but not in HC. Accounting for exercise intensity (HR) did not alter the observed lactate responses following the 12MWT. Taken together, these findings indicate that plasma lactate, as assessed under the present test conditions, has limited value for distinguishing MM patients from HC. It is possible that different results might have been obtained if exercise intensity had been standardized using individualized or relative workload approaches (e.g., HR–guided protocols).

### 4.4 Limitations of Fixed Workload Exercise Tests

Multiple attempts have been made to develop diagnostic exercise tests with a fixed workload for MM. Finsterer et al. conducted several studies [10, 11, 12, 13] using a 15‐min cycling protocol at a fixed absolute workload of 30 watts, reporting sensitivities and specificities of 66%–88% and 84%–94%, respectively, when comparing MM patients with HC or other neurological disorder controls. However, not all MM patients are able to sustain a 30 watts workload protocol [16]. A similar limitation would apply to walk tests using a fixed treadmill pace, as some patients would be unable to maintain the speed while others would be insufficiently challenged. In the present study, the shortest walking distance during the 12MWT was 306 m, illustrating the wide variability in functional capacity among patients. These observations suggest that relative workload protocols, adjusted to individual exercise capacity, may be more appropriate for MM. Finsterer et al. [10] also included a comparison with relative workload (30% of maximal capacity). Although they reported higher diagnostic sensitivity for absolute workload protocols, the study was limited by small sample size, heterogeneity of MM phenotypes, and limited adjustment for physiological factors such as HR as an indicator of exercise intensity. This highlights a general challenge in standardizing exercise intensity using relative workload approaches. While HR–guided protocols may offer a more direct reflection of physiological strain, this remains speculative and warrants further investigation. Furthermore, conditions such as ischemic heart disease, anemia, lung disease, and claudication may elevate HR and plasma lactate at low workloads, thereby reducing the specificity of exercise‐based tests. Overall, heterogeneity in exercise capacity and physiological responses limits the diagnostic utility of exercise‐based testing in MM, irrespective of whether fixed or relative workload protocols are applied.

### 4.5 Outliers

The two outliers (patients #4 and #11) showed plasma lactate values markedly higher than the rest of the cohort at end test (9.7 and 14.6 mmol/L, respectively), despite walking distances that were within the overall range observed across participants. As highlighted in Table 1, they were unrelated men with different genotypes and phenotypes. Patient #4 with chronic progressive external ophthalmoplegia (CPEO) due to a large‐scale deletion had 65% mutation load in muscle, whereas patient #11 with m.5133_5134delAA variant carried a 90%–92% mutation load in muscle. The m.5133_5134delAA variant likely causes a more global defect in mitochondrial translation and thus a more severe diffuse myopathy phenotype. Although elevated mutation load may contribute to impaired oxidative phosphorylation and increased reliance on anaerobic metabolism, the present data do not support a direct one‐to‐one relationship between mutation load and lactate response. Mutation load likely represents an important contributing factor, but it alone cannot explain the observed variability, suggesting that additional physiological or disease‐related factors may also influence exercise‐induced lactate responses.

### 4.6 Systemic versus Local Biomarkers

Beyond differences in exercise intensity, another factor limiting the diagnostic value of exercise‐based biomarkers is the distinction between systemic and local metabolic measurements. Currently, the most sensitive tests for MM assess impaired oxygen extraction within the working muscle during single‐extremity exercise using near‐infrared spectroscopy or arteriovenous oxygen differences [4, 5, 17]. In the present study, measured systemic venous oxygenation during and after the walk tests were deemed of no use. Because mitochondrial dysfunction primarily affects oxygen use within the muscle, systemic measurements cannot capture these local abnormalities. Consistent with this, oxygen did not differ between MM patients and HC. Plasma lactate, while practical to measure, may also have limited ability to reflect local mitochondrial defects.

### 4.7 Additional Findings and Clinical Implications

The original motivation for exercise‐based screening tests was to identify a simpler and more accessible diagnostic screening tool than invasive procedures such as muscle biopsy or more complex metabolic investigations. However, based on previous evidence and the findings of the present study, plasma lactate responses in such tests do not provide sufficient diagnostic discrimination to justify their use. In the era of modern molecular genetic diagnostics, these tests are therefore unlikely to play a meaningful role in diagnosing MM. Instead, exercise testing may be more valuable for evaluating patients with established MM. Plasma lactate has been shown to serve as a useful biomarker for treatment effects, as reductions at constant submaximal exercise indicate improvements in mitochondrial oxidative capacity [17]. In this regard, our findings contribute additional practical considerations for the interpretation of lactate measurements. Specifically, the difference in plasma lactate concentrations in both MM and HC during fasting compared with after a standardized meal was highly significant (*p* < 0.001). This shows that nutritional status can substantially influence lactate measurements and should therefore be carefully standardized in future studies. We recommend standardization of pre‐test nutritional conditions, including fasting status, timing of the last meal, and/or the use of a standardized pre‐test meal with defined composition. Our results also provide insight into the physiological responses of MM patients during walking exercise. MM patients accumulated significantly more lactate following the 12MWT than after the 6MWT, whereas no such difference was observed in HC. This suggests that the longer walking duration may impose a greater metabolic challenge in MM patients, even though the test did not allow diagnostic discrimination between groups. Variability in walking distance among MM patients reflected differences in exercise tolerance, likely related to disease severity and phenotypic heterogeneity. Walk tests are already used as functional outcome measures in mitochondrial disease, with the 6MWT frequently applied [17, 18, 19, 20, 21]. Our findings suggest that longer protocols, such as the 12MWT, may provide additional physiological information and complement existing outcome measures. Despite potential learning effects, 12MWT results were consistent across two test days, indicating reproducibility. Further research is however needed to establish the validity, reliability, and clinical relevance of the 12MWT as an outcome measure for monitoring disease progression or treatment effects in MM.

### 4.8 Strengths and Limitations

This study has several limitations. Although we included a substantial number of patients with MM relative to disease frequency, the sample size may still limit the generalizability of the findings. Furthermore, variability in phenotype and genotype was not accounted for. While the study investigates the 12MWT and plasma lactate as a biomarker, it does not delve deeply into direct assessments of mitochondrial function.

The study also has strengths. It employed a comprehensive approach, evaluating the effects of both dietary and exercise interventions on plasma lactate in MM patients and comparing outcomes to HC. The protocol is based on tests that are accessible and practical in clinical application, since it is low‐cost and low equipment. Furthermore, it evaluates parameters that directly reflect the daily functional impairments MM patients might experience.

## 5 Conclusion

Changes in plasma lactate are not diagnostically useful for MM in a 12‐min submaximal walk test or during a 20‐min post‐exercise recovery period. Lactate levels were significantly influenced by nutritional status, and longer walking durations revealed greater lactate accumulation in MM patients, reflecting physiological differences and variability in exercise tolerance. Despite its limited diagnostic value, the 12MWT may serve as a functional outcome measure in clinical trials or longitudinal studies. Further studies are however needed to establish the validity and reliability of the 12MWT as an outcome measure.

## Author Contributions

**Christine Lando:** formal analysis, data curation, writing – original draft, writing – review and editing, validation, visualization. **Tahmina Khawajazada:** investigation, data curation, writing – review and editing. **Jesper Helbo Storgaard:** investigation, data curation, writing – review and editing. **Nanna Scharff Poulsen:** formal analysis, writing – review and editing, visualization. **John Vissing:** methodology, conceptualization, resources, supervision, validation, writing – review and editing, funding acquisition, project administration. **Nicoline Løkken:** data curation, methodology, conceptualization, investigation, validation, writing – review and editing, supervision, funding acquisition, project administration.

## Funding

This work was supported by Aase Ejnar Danielsen foundation [10‐002136], Augustinus foundation [17‐4215], and Else og Mogens Wedell‐Wedellsborgs foundation [6‐18‐1]. The authors confirm independence from the funding sources; the content of the article has not been influenced by the funding sources.

## Ethics Statement

All procedures followed were in accordance with the ethical standards of the responsible committee on human experimentation (institutional and national) and with the Helsinki Declaration of 1975, as revised in 2000. Informed written consent was obtained from all patients before being included in the study. The study was approved by the ethics committee of Copenhagen (H‐17032512) and listed on clinical trials.gov (NCT03513835) prior to inclusion.

## Conflicts of Interest

The authors declare no conflicts of interest.

## Data Availability

The data that support the findings of this study are available on request from the corresponding author. The data are not publicly available due to privacy or ethical restrictions.
